# Integrated analysis of transcriptome and metabolome of *Arabidopsisalbino or pale green* mutants with disrupted nuclear-encoded chloroplast proteins

**DOI:** 10.1007/s11103-014-0194-9

**Published:** 2014-05-03

**Authors:** Masakazu Satou, Harumi Enoki, Akira Oikawa, Daisaku Ohta, Kazunori Saito, Takushi Hachiya, Hitoshi Sakakibara, Miyako Kusano, Atsushi Fukushima, Kazuki Saito, Masatomo Kobayashi, Noriko Nagata, Fumiyoshi Myouga, Kazuo Shinozaki, Reiko Motohashi

**Affiliations:** 1 Plant Science Center (Center for Sustainable Resource Science), RIKEN, Yokohama, Kanagawa 230-0045 Japan; 2 Graduate School of Agricultural and Biological Science, Osaka Prefecture University, Sakai, Osaka 599-8531 Japan; 3 K.K., Bruker Daltonics, Yokohama, Kanagawa 221-0022 Japan; 4 Department of Molecular Biology and Biotechnology, Graduate School of Pharmaceutical Sciences, Chiba University, Chiba, Chiba 263-8522 Japan; 5 Department of Agriculture, Shizuoka University, 836 Ohoya Suruga-ku, Shizuoka, Shizuoka 422-8529 Japan; 6 Present Address: Dragon Genomics Center, TAKARA BIO INC., Ootsu, Shiga 520-2198 Japan; 7 BioResource Center, RIKEN, Tsukuba, Ibaraki 305-0074 Japan; 8 Faculty of Science, Japan Woman’s University, Bunkyou-ku, Tokyo, 112-8681 Japan

**Keywords:** Albino or pale-green, *Arabidopsis thaliana*, Chloroplast, Metabolome, Transcriptome, Nitrogen assimilation

## Abstract

We used four mutants having albino or pale green phenotypes with disrupted nuclear-encoded chloroplast proteins to analyze the regulatory system of metabolites in chloroplast. We performed an integrated analyses of transcriptomes and metabolomes of the four mutants. Transcriptome analysis was carried out using the Agilent Arabidopsis 2 Oligo Microarray, and metabolome analysis with two mass spectrometers; a direct-infusion Fourier transform ion cyclotron resonance mass spectrometer (FT-ICR/MS) and a gas chromatograph-time of flight mass spectrometer. Among approximately 200 known metabolites detected by the FT-ICR/MS, 71 metabolites showed significant changes in the mutants when compared with controls (*Ds* donor plants). Significant accumulation of several amino acids (glutamine, glutamate and asparagine) was observed in the albino and pale green mutants. Transcriptome analysis revealed altered expressions of genes in several metabolic pathways. For example, genes involved in the tricarboxylic acid cycle, the oxidative pentose phosphate pathway, and the de novo purine nucleotide biosynthetic pathway were up-regulated. These results suggest that nitrogen assimilation is constitutively promoted in the albino and pale green mutants. The accumulation of ammonium ions in the albino and pale green mutants was consistently higher than in *Ds* donor lines. Furthermore, genes related to pyridoxin accumulation and the de novo purine nucleotide biosynthetic pathway were up-regulated, which may have occurred as a result of the accumulation of glutamine in the albino and pale green mutants. The difference in metabolic profiles seems to be correlated with the disruption of chloroplast internal membrane structures in the mutants. In albino mutants, the alteration of metabolites accumulation and genes expression is stronger than pale green mutants.

**Electronic supplementary material:**

The online version of this article (doi:10.1007/s11103-014-0194-9) contains supplementary material, which is available to authorized users.

## Introduction

In the post genome-sequencing era, functional analyses of identified genes have been performed systematically by using various mutant collections. Transcriptome analyses have provided us systematic characterization of gene expression profiles not only in various tissues and organs but also under various growth conditions. Proteome analyses have revealed various novel proteins not only in various tissues and organelle but also under various environmental conditions. Recently, technology development in mass spectrometry has improved its reliability, sensitivity of detection and measurement speed in comprehensive analyses of whole metabolites (Sumner et al. 2003; Villas-Bôas et al. 2005; Hall 2006) and whole proteins (proteome) (Kersten et al. 2002; Baginsky and Gruissem 2006; Giacomelli et al. 2006). More recently there have been reports which include the integrated analysis of transcriptome and metabolome data (Caldana et al. 2011; Fortes et al. 2011) as well as research regarding the transcriptomic, proteomic and metabolomic analysis of maize response to UV-B (Casati et al. 2011a, b). Tools have also been developed for the joined visualization of transcriptomics and metabolomics (Wägele et al. 2012).

An organism has the ability to acquire tolerance to external stresses such as light, nutrient starvation and cold. Internal biological networks including gene expression and cellular signal transduction are regulated in response to these external environmental changes, which results in an accumulation of stress-specific metabolites. Such accumulations of metabolites may cause phenotypic changes. An abundance of transcripts measured by microarray does not always correlate with an abundance of proteins (Gygi et al. 1999) and their enzymatic activities (Sumner et al. 2003). Thus expression profiles (of transcriptome and proteome) in mutants are not always consistent with biochemical and metabolic phenotypes. However, metabolite distribution in the organism is the final product of gene expression, which is directly connected to biochemical phenotype. Therefore, metabolome analyses can provide insights into novel gene functions that cannot be obtained with a conventional morphological analysis of various mutants. In particular, metabolome analyses have revealed silent mutant phenotypes that are analogous to those of wild-type (Weckwerth et al. 2004), and have predicted gene functions from classification of mutants with the same phenotype, a process known as metabolic fingerprinting.

We previously isolated *albino or pale*-*green* (*apg*) mutants of *Arabidopsis thaliana*. The mutations resulted from disruption of nuclear-encoded chloroplast proteins by the insertion of the *Ds* transposon. Since there were various phenotypes of these *apg* mutants, metabolic regulation in chloroplast contained several metabolic cascades. Active biosynthesis of various metabolites is carried out in chloroplast. Most of the gene products involved in metabolic pathways are encoded in cell nuclei and transported into chloroplast to perform various functions. As the first step to identify regulatory systems of metabolites in chloroplast, we thought it important to show how metabolite profiles are altered in the *apg* mutants. This study was designed to use integrated analyses of transcriptome and metabolome of four mutants: *apg2* (*albino or pale green 2*) (Motohashi et al. 2001); *apg3* (*albino or pale green 3*) (Motohashi et al. 2007); *cla1* (*cloroplastos alterados1*) (Mandel et al. 1996; Estévez et al. 2000); and *ch42* (*chlorina locus 42*) (Rissler et al. 2002).

We performed transcriptome analysis by using the Agilent Arabidopsis 2 Oligo Microarray (V2), and metabolome analysis by using two mass spectrometers; Apex-q70e with Apollo II ESI (electron spray ionization) source FT-ICR/MS (Fourier transform ion cyclotron resonance mass spectrometer) and GC-TOF/MS (gas chromatograph-time of flight mass spectrometer). Plant metabolome analyses were performed using various mass spectrometers, namely GC/MS (Kaplan et al. 2004; Kolbe et al. 2006), and FT-ICR/MS (Aharoni et al. 2002; Mungur et al. 2005; Tohge et al. 2005; Oikawa et al. 2006; Nakamura et al. 2007). Mass spectrometers have different advantages depending on their type. The GC/MS is a traditional mass spectrometer, the GC device of which can separate complex biological mixtures. It is effective for the analysis of semi-volatile organic compounds such as sugars, organic acids, amino acids, sugar alcohols, amines and amides. Unlike other mass spectrometers, the advantages of FT-ICR/MS are its high-resolution and ability to determine exact mass in order to predict chemical formulae. We used direct infusion FT-ICR/MS with ESI, which is known as a soft ionization method. In this study we report an integrated analyses of the transcriptomes and metabolomes of four mutants to compare their biochemical properties, phenotypes, metabolic profiles and related gene expressions.

## Results

### Characteristics of Albino and pale green Arabidopsis mutants

**Fig. 1:**
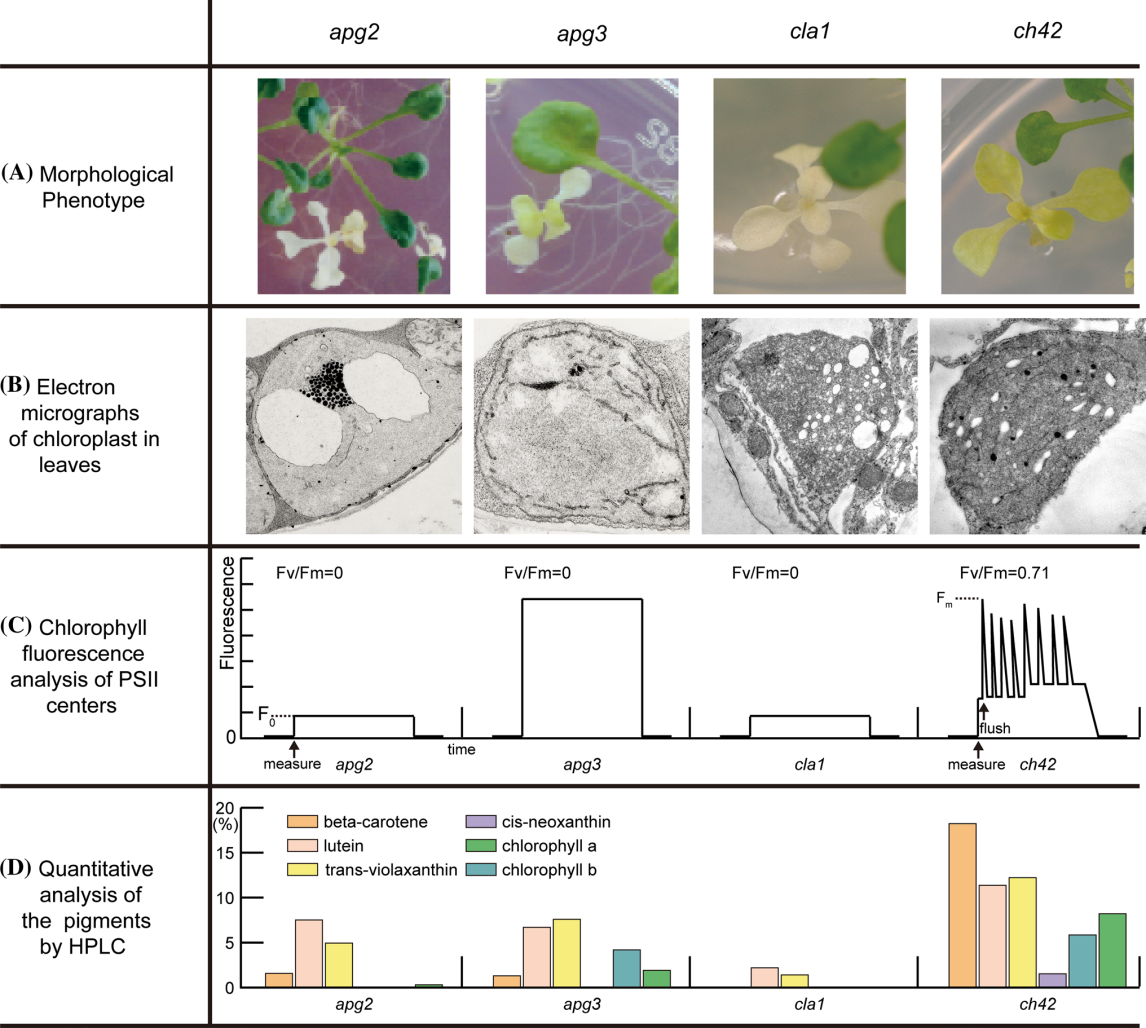
Summary of four *apg* mutants’ phenotypes that have been reported previously. **a** Morphological phenotype of the mutants that were grown for 21 days on GM medium containing 1 % sucrose. **b** Electron micrographs of leaf chloroplasts. **c** Schemes of chlorophyll fluorescence profiles measured by PAM. The values in each left top panel show Fv/Fm (=FmFo/Fm) indicating the state of PSII. **d** *Bar plots* show the relative abundance of five pigments compared with that of wild-type measured by HPLC

As mentioned in the introduction, we selected four albino and pale green mutants for this study: *apg2* (Motohashi et al. 2001); *apg3* (Motohashi et al. 2007); *cla1* (Mandel et al. 1996; Estévez et al. 2000); and *ch42* (Rissler et al. 2002). Descriptions of the mutations in the four mutants are as follows. The *apg2* mutant is disrupted in a TatC homologue of the *Escherichia coli* delta-pH dependent protein transporter (Motohashi et al. 2001). The *apg3* mutant is disrupted in a gene homologous to a eukaryotic ribosome release factor (RF1) and thus *APG3* operates as a ribosome release factor in chloroplast (Motohashi et al. 2007). The gene disrupted in the *cla1* mutant encodes the 1-deoxy-d-xylulose-5-phosphate (DXP) synthase, which is an enzyme upstream of isoprenoid biosynthesis (Mandel et al. 1996; Estévez et al. 2000). The mutation in the *ch42* mutant is in the protoporphyrin IX chelatase subunit CHLI1 that functions in chlorophyll biosynthesis (Rissler et al. 2002). The gene loci references according to TAIR (version 10) are as follows: *APG2* gene, AT2G01110; *APG3* gene, AT3G62910; *CLA1* gene, AT4G15560; *CHLI1* gene, AT4G18480. The phenotypes of the *apg2* and *cla1* mutants are albino, whereas the *apg3* mutant is a pale-green, and the *ch42* mutant is a yellowish pale-green (Fig. 1a). In previous research, it has been reported that the chloroplast internal membrane structures have been deficient in all 4 of the mutants (Fig. 1b) (Motohashi et al. 2001; 2007, Mandel et al. 1996; Estévez et al. 2000; Rissler et al. 2002). The plastids of the *apg2* and *cla1* mutants did not contain thylakoid membranes, while those of *apg3* and *ch42* mutants contained immature thylakoid membranes. The F_v_/F_m_ value measured by pulse amplitude modulated (PAM) fluorometer showed a potential quantum yield of photosystem II (Krause et al. 1988). That value in a 3-week old *Ds* donor plant is approximately 0.72; however the values in the three mutants *apg2*, *cla1*, and *apg3* were nearly 0 (Fig. 1c). In contrast, the F_v_/F_m_ value in the mutant *ch42* was approximately 0.71 (Fig. 1c). HPLC analyses showed that amounts of various pigments (chlorophyll *a*, chlorophyll *b*, cis-neoxanthin, trans-violaxanthin, lutein, and beta-carotene) in each of the mutants were reduced to 0–20 % of that of *Ds* donor plants (Fig. 1d).

### Analysis of the metabolic phenotypes of albino and pale green mutants versus Ds donor plants using direct-infusion ESI FT-ICR/MS

Using direct-infusion ESI FT-ICR/MS, we detected more than 2,500 independent ion peaks in every spectral scan of each sample, irrespective of experimental setting with different extraction solvents and two ionization operation modes (positive and negative). The negative operation mode was usually characterized by larger numbers of detectable peaks compared with the positive mode, and total peak numbers from *Ds* donor plants were larger than those from the mutants.

Almost 40 % of those 2500 peaks were detected at very low intensities (close to background levels), and such peaks were not consistently reproduced in subsequent experiments. We also evaluated the performance characteristics of direct-infusion ESI FT-ICR/MS for detection and quantification of the four mutants. We made the following four kinds of comparisons of peak intensity values found in spectral scan data. First, we checked the consistency in results of MS analysis by extracting one sample and analyzing it twice. Second, to examine the difference that occurs during extraction, we extracted the same sample twice and analyzed it. Third, we made the same comparison as the second but this time using methanol and acetone extraction. Fourth and finally, we extracted and analyzed completely different samples. Data spread was greater in the third and fourth comparisons than in those of the first and second. After performing these various tests we decided that direct-infusion ESI FT-ICR/MS was a suitable method to use for the metabolic phenotyping of the four mutant plants in our study. Four scatter plots and detailed descriptions of each are shown in Supplemental Figure 1.

### Metabolomics profiling using principal component analysis and *t* test

**Fig. 2:**
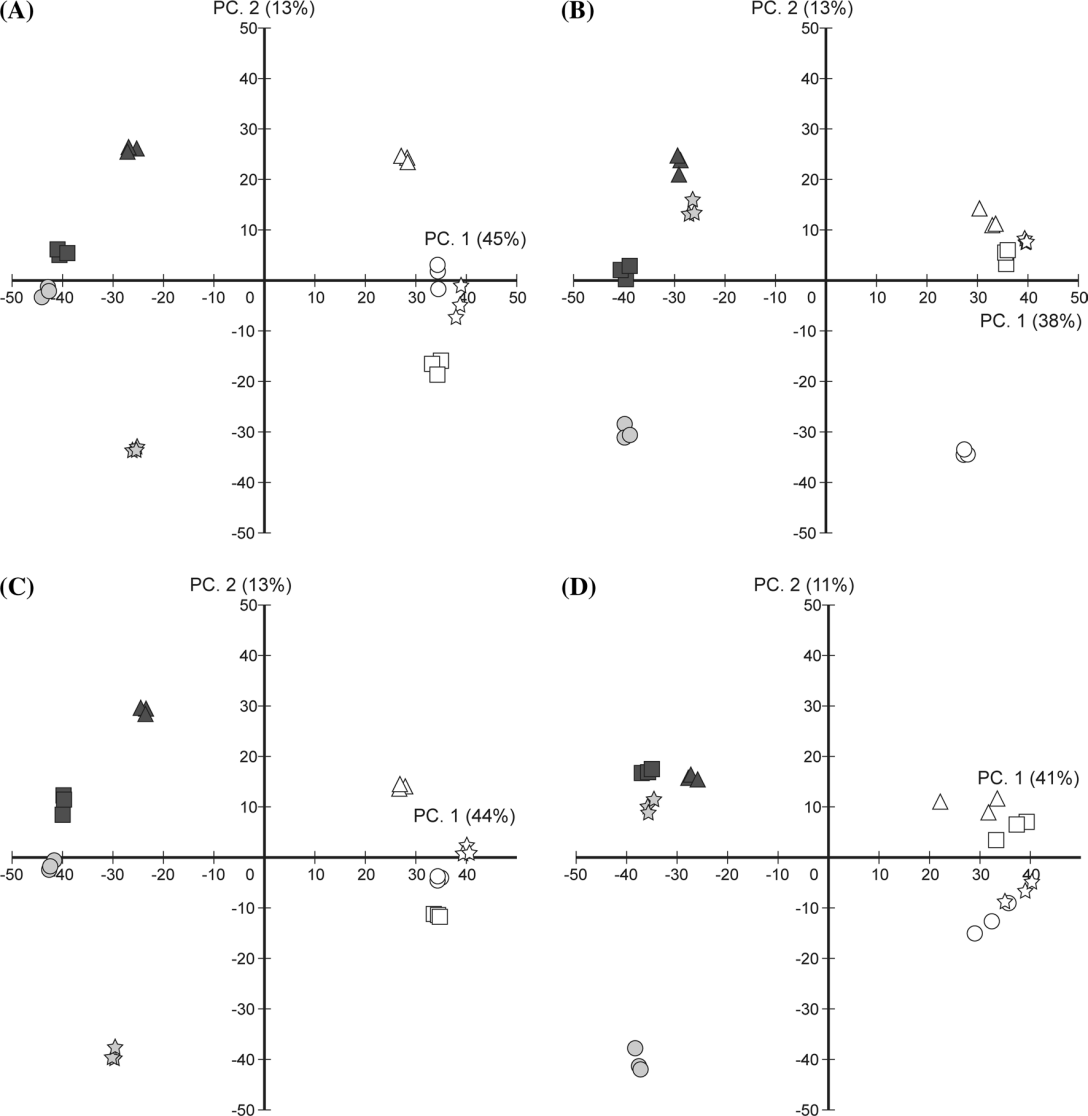
Biplot of Principal Component Analysis (PCA) applied for ESI-FT-ICR/MS. X-axis and Y-axis are principal component 1 (PC1) and principal component 2 (PC2), respectively. *Solid circle* is the *apg2* mutant, *unfilled circle* is the *apg2* *Ds* donor line, solid square is the *apg3* mutant, unfilled square is the *apg3* *Ds* donor line, *solid star* is the *cla1* mutant, *unfilled star* is *cla1* *Ds* donor line, *solid triangle* is the *ch42* mutant, and *unfilled triangle* is the *ch42* *Ds* donor line. *Gray colored symbol* is albino phenotype, *black* is *pale-green* phenotype. **a** Methanol extraction, positive charge mode measurement. **b** Methanol extraction, negative charge mode measurement. **c** Acetone extraction, positive charge mode measurement. **d** Acetone extraction, negative charge mode measurement.

Principal components analysis (PCA) is a technique used to reduce the dimensionality of multivariate data. We projected our data to two principal components. The first principal component (PC1) shows a clear difference between the mutants and the *Ds* donor plants (Fig. 2). The contribution ratio of PC1 was approximately 40–45 % in each matrix, which may reflect a vast difference of metabolites between the mutants and the *Ds* donor plants. PC2 separates the mutants, which had similar data patterns under different extraction solvent conditions (Fig. 2a, c), but differing data patterns across positive and negative ionization operation modes (Fig. 2c, d). For example the *cla1* mutant was plotted far from the other mutants under positive mode, and the *apg2* mutant was plotted far from the other mutants under negative mode.

To estimate the volume of the metabolites from the obtained ion peaks, we used Welch’s t-tests on the mutant values and the *Ds* donor line quantitative values as described in “Materials and methods”. When we searched manually for candidate compounds based on empirical formulas, we found approximately 150–200 peaks corresponding to known metabolites. Peaks that showed significant statistical difference between mutants and *Ds* donor lines (FDR < 0.05) were selected, corresponding to a total of 71 compounds (Supplemental Table 1). As follows, a short list of the compounds and their difference in relative abundance between mutants and their *Ds* donor lines: in the mutants there was an increase in five amino acids (asparagine, glutamine, glutamate, arginine, and histidine) and two organic acids (citrulline and glucarate), an increase in vitamin B6 and its derivatives, and a decrease in ascorbate and monodehydroascorbate (Supplemental Table 1). Phenylpropanoids were fewer for the most part, but levels of sinapoyl-glucose and bis-sinapoyl-glucoside were found to be increased in the *cla1* mutant. There was also a reduction in nine terpenoids including carotenoids, but a relative increase was found in monoterpene (6-endo-Hydroxycineole) and sesquiterpene (copaene) in the *apg2* and *cla1* mutants. It is noteworthy that intermediate metabolites of de novo purine biosynthesis were affected differently among the mutants (Supplemental Table 1; Fig. 5; Supplemental Fig. 3). Nine flavonoids were detected. In particular, levels of flavonol glycosides such as cyanidin-rhamnosylglucoside, kaempferol-galactoside-rhamnoside and kaempferol-sophoroside were greater in the mutants. Among the sugars there were fewer pentoses and septuloses (sedoheptulose) and an increased number of hexoses, but flavonol and sinapoyl-glucose levels were distinctly different among the mutants. For example there were very high levels of flavonol and sinapoyl-glucose accumulated in the *cla1* mutant in positive ion mode (Fig. 2a, c). Although the *apg2* mutant differed from the other mutants on the PC2 axis in negative ion mode (Fig. 2b, d), we could not detect obvious *apg2*-specific metabolites, due to similar profiles between the *apg2* mutant and its *Ds* donor control plant (*Ds*389-13). It might be because in *Ds*389-13, the *Ds* transposon has been inserted into the gene locus AT5G66210 [which encodes a calcium-dependent protein kinase (Ito et al. 2002)] before the *APG2* gene was inserted.

### Parallel analysis of amino acids and sugar metabolites using GC-TOF/MS

It is difficult to identify compounds that have the same mass when using direct-infusion FT-ICR/MS. For this reason we also used GC-TOF/MS to analyze compounds. Six sample pools were created from each mutant and *Ds* donor plant. In a manner similar to FT-ICR/MS, log ratios were calculated and Welch’s t-tests were performed. As a result, the 68 compounds that were statistically significant (FDR < 0.05) were identified in at least one out of four experiments. Fourteen of those compounds, tyrosine, tryptophan, asparagine, arginine, glutamate, histidine, pyroglutamate, glutamine, fructose, glucose, glycerol 3-phosphate, alpha-tocopherol, linoleate and stearate were also detected in FT-ICR/MS, and their relative abundances were similar. The polyamines putrescine and spermidine were also accumulated in the mutants (Supplemental Table 2). The hexoses that accumulated in the mutants (shown by FT-ICR/MS) were fructose and glucose, whereas myo-inositol levels were decreased.

When performing metabolomics profiling analysis using either FT-ICR/MS or GC-TOF/MS, we observe an increase of several amino acids. Moreover, results of previously performed proteome analyses suggest the accumulation of proteins related to amino acid metabolism is a common characteristic in albino mutants (Motohashi et al. 2012). Activation of amino acid synthesis or metabolism is considered to be a feature common to heterotrophic metabolism caused by an albino mutation.

### Transcriptomics profiling of microarrays using hierarchical clustering and functional categorization based on Gene Ontology Slim terms

A two-color microarray was used for transcriptome analysis. Intensity log ratio was calculated from mutant/*Ds* donor (see Materials and methods). The 2,812 significantly altered genes were chosen where FDR was <10^−4^ (Supplemental Table 3).

**Fig. 3:**
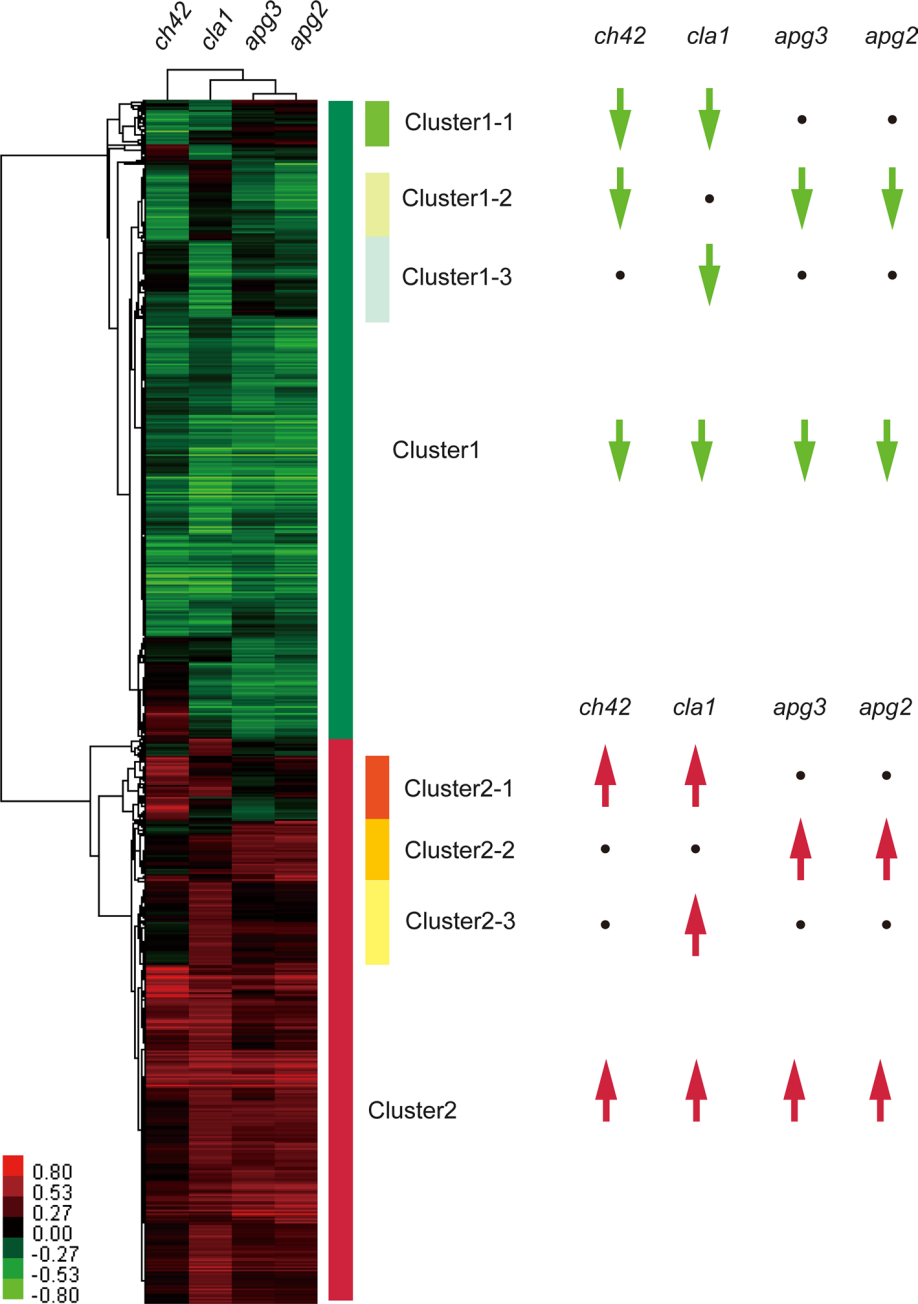
GeneTree of Hierarchical Cluster Analysis applied for microarray gene expression profiles. The GeneTree was calculated from 2,812 significantly altered genes (FDR < 1^−4^). Gene expression in the mutants higher and lower than those of the *Ds* donor lines are shown in *red* and *green*, respectively. Eight clusters were chosen based on their expression and their profiles were displayed as *arrow symbols* on the right panel. The altered genes were divided between 1,490 up-regulated genes as cluster1, and 1,322 down-regulated genes as cluster2. We extracted three subclusters from each of cluster1 and cluster2. Cluster1-1 (*ch42* down-regulated, *cla1* down-regulated, *apg3* not changed, *apg2* not changed), cluster1-2 (*ch42* down-regulated, *cla1* not changed, *apg3* down-regulated, *apg2* down-regulated), cluster1-3 (*ch42* not changed, *cla1* down-regulated, *apg3* not changed, *apg2* not changed) and cluster2-1 (*ch42* up-regulated, *cla1* up-regulated, *apg3* not changed, *apg2* not changed), cluster2-2 (*ch42* not changed, *cla1* not changed, *apg3* up-regulated, *apg2* up-regulated), cluster2-3 (*ch42* not changed, *cla1* up-regulated, *apg3* not changed, *apg2* not changed) were extracted from the two clusters

By hierarchical cluster analysis, we obtained eight clusters that showed similar gene expression profiles among the four mutants (Fig. 3). We then annotated these clusters by biological classification using Gene Ontology and GO Slim.

**Fig. 4:**
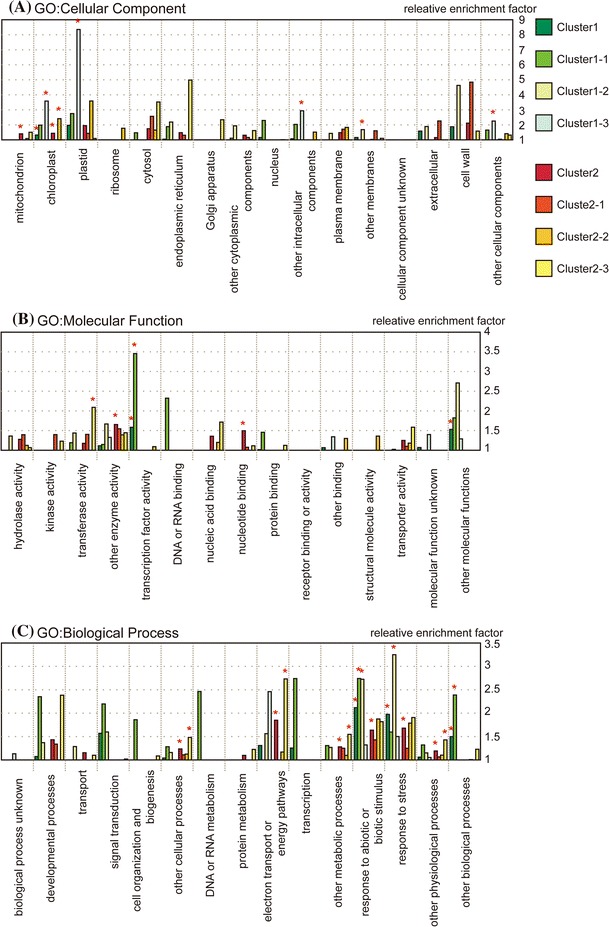
Functional classification of gene clusters based on GO Slim. *Bar* plot represents the relative enrichment factor that is the odds ratio of GO class corresponding genes in a cluster, compared with those in overall *Arabidopsis*. Asterisked GO classes were statistically significant (FDR < 10^−4^) in each of the three GO categories **a** Cellular Component, **b** Molecular Function, **c** Biological Process

To determine whether consensus features of genes were contained in each cluster, we first annotated them by biological classification based on Gene Ontology (GO) provided by the Gene Ontology Consortium (http://www.geneontology.org/). We totaled the corresponding genes for each GO term, and then using Fisher’s exact test, screened to find GO terms within each cluster that had statistically higher relative enrichment factors in respect to *Ds* donor plants (Supplemental Table 3). In addition, we also referenced GO Slim provided by TAIR (Rhee et al. 2003), as GO terms alone are in many cases too specific to grasp an appropriate overview of a mutants’ characteristics (Fig. 4).

‘Chloroplast’ appeared to be the most enriched class among almost all clusters. This suggests that the expression of nuclear-encoded chloroplast protein genes was affected greatly. An enrichment analysis of Cluster2 (composed of up-regulated genes over each of the four mutants) indicated the activation of genes related to nucleoside monophosphate metabolism (including purine nucleoside), and of genes related to the metabolism of nitrogen compounds, such as amino acids and amine. This is in agreement with metabolome results gained via FT-ICR/MS described above.

Under the classification for ‘Cellular Component’ we can see that genes related to plastids were highly active in Cluster1-3. In Cluster1-3 the *cla1* mutant down-regulated the most, which suggests that genes related to plastids that were not as repressed in the other mutants were more strongly repressed in the *cla1* mutant. Detailed classification with GO terms revealed that Cluster1-3 contained genes related to ‘chlorophyll biosynthesis’, ‘porphyrin biosynthesis’ and ‘photosynthesis, light reaction’ (Supplemental Table 4). From this we can deduce that the *cla1* mutant specifically repressed genes that are considered to be photosynthesis-related genes, such as genes encoding chlorophyll biosynthesis.

Under “Molecular Function” the following genes were enriched: ‘other enzyme activity’ and ‘nucleotide binding’ in Cluster2, ‘transferase activity’ in Cluster2-3, and ‘transcription factor activity’ in Cluster1 and Cluster1-2 (Fig. 4b). Detailed classification of genes using GO terms in Cluster2-3 revealed up-regulated genes mainly in the *cla1* mutant, related to glycosyl- or acyl-transfer enzymes. It should be noted that Cluster2-3 contained six UDP-glucosyl transferase genes. UDP-glucosyl transferase belongs to glycosyltransferase family 1, which uses phenylpropanoids such as flavonol and sinapate as substrates (Rohde et al. 2004) (http://afmb.cnrs-mrs.fr/CAZY/). Not coincidentally our data shows that sinapoyl-glucose, bis-sinapoyl-glucoside and flavonol were all accumulated in the *cla1* mutant (Supplemental Table 1).

Finally under ‘Biological Process’, two GO classes ‘response to abiotic or biotic stimulus’ and ‘response to stress’ were enriched (Fig. 4c). The genes belonging to these classes are redundant, however it is worthy to note that ‘response to abiotic or biotic stimulus’ contains genes related to endogenous stimuli, such as sugars and hormones. These two classes were enriched not only in Cluster1 but also in Cluster2, which indicates that the mutants have suffered from various stimuli and stresses. The same accumulation could be found via proteome analysis as well (Motohashi et al. 2012). It is clear that responses to stress are active in albino mutants, likely due to their being weak from various stresses. In these GO classes, up-regulated genes were glutathione S-transferase, LEA (late embryogenesis abundant) proteins, peroxidase, catalase, heat shock proteins and superoxide dismutase, all of which are induced by oxidative stress. In contrast, down-regulated genes were those affected by a variety of stresses, including oxidative stress. In particular, the repressed genes were auxin-responsive proteins and cold responsive proteins such as *cor47*, *cor15a* and *cor15b*. The GO class ‘electron transport or energy pathway’ was enriched in Cluster2 and Cluster2-3. This class represents genes involved in reduction and energy liberation pathways. Typically, these genes are related to production of energy compounds such as ATP and NADPH. Detailed classification with GO terms revealed the GO term ‘main pathways of carbohydrate metabolism’ and its children terms ‘hexose catabolism’ and ‘glycolysis’ were enriched in Cluster2-3 (Supplemental Table 4). Gene expression related to metabolic pathways, such as the TCA cycle, glycolysis, and the oxidative pentose phosphate pathway were up-regulated among the mutants, in particular the *cla1* mutant.

## Discussion

### An overview of the metabolome and transcriptome in albino and pale green mutants

In this study we observed that nitrogen assimilation was promoted in all four mutants, but this was achieved in different ways. One was the up-regulation of several genes related to the nitrate assimilation pathway. The other was accumulation of glutamine and glutamate synthesized from ketoglutarate via the GS/GOGAT cycle, and asparagine synthesized by asparagine synthetase. These three amino acids are compounds that play a role in nitrogen storage and transport (Lea and Mifflin 2003). TCA (Tricarboxylic acid) cycle and OPPP (Oxidative pentose phosphate pathway) genes were highly expressed in the mutants; the former is for ketoglutarate synthesis and the latter is for providing reducing power to nitrate assimilation (Emes and Neuhaus 1997). In addition, the gene expressions of enzymes associated with glycolysis and phenylpropanoid biosynthesis pathways were affected. This correlated with metabolomic profiles and the degree of disruption of the chloroplast inner membrane. We also detected the alteration of intermediate metabolites in the de novo purine nucleotide biosynthesis pathway, and the accumulation of pyridoxine and its derivatives. As both of these phenomena require glutamine amides for initiation, it is suggested that the increase in glutamine accumulation activates the down-stream events of these pathways.

There is a correlation between the degree of disruption of the chloroplast inner membrane and the degree of accumulation of the metabolomics profiles mentioned above. In albino mutants, the alteration of metabolite accumulation and gene expression is stronger than in pale green mutants. We also detected the alteration of intermediate metabolites in de novo purine nucleotide biosynthesis pathways. Because this pathway requires glutamine amides for initiation, it is suggested that strongly accumulated glutamine activates the down-stream events of these pathways.

In plants, there are relationships between carbohydrates such as sugars and the regulation of glutamine synthesis (Oliveira and Coruzzi 1999). Endogenous accumulation of glutamine represses the transcription of the nitrate and nitrite reductase genes (Vincentz et al. 1993; Hoff et al. 1994). In contrast, when sugars are fed to starved leaves, transcripts of nitrate reductase increase (Vincentz et al. 1993). In this study we found that glutamine was accumulated in the mutants (Supplemental Table 1; Supplemental Table 2). However, the transcripts of nitrate reductase were also up-regulated (Supplemental Table 5; Fig. 3). The reason may be that the accumulation of sugars such as glucose and fructose overrides the regulation of nitrate reductase genes (Klein et al. 2000).

The reason for the activation of nitrogen assimilation in the albino and pale green mutants is uncertain, but it is possibly associated with increasing endogenous ammonium. We measured endogenous ammonium levels in the mutants. The accumulation of ammonium ions in the mutants was consistently higher than in their *Ds* donor lines (Supplemental Figure 2). We thought that the reason for the degradation of photosynthetic proteins such as ribulose-1,5-bisphosphate carboxylase/oxygenase (Rubisco) and the chlorophyll a/b binding protein (Lhcp) was because the mediums of grown albino and pale green mutants and *Ds* donor lines had similar ammonium ion content (data not shown).

Extensive protein degradation leads to a drastic increase of internal ammonium content (Feng and Barker 1992). Even though the photosynthetic genes of the mutants tend to be repressed, expressions of Rubisco and Lhcp were higher than that of the other genes, thus some translated proteins of these genes have been imported into chloroplasts. Lhcp binds pigments in chloroplasts, which are then protected against protein degradation (White and Green 1987). However, chlorophyll content was low in the mutants (Fig. 1), thus almost all Lhcp could be degraded due to it being unable to bind to chlorophyll. It has been reported that the degradation of Rubisco proteins is triggered by ROS (Ishida et al. 1998). The ascorbate–glutathione cycle has the ability to remove ROS that are generated in tissue, but the cycle was not activated in this case because of the reduction of ascorbate (del Ri´o et al. 1998). For this reason, a large amount of ROS induced Rubisco degradation in the mutants. It is possible also that in order to compensate for the degradation of Lhcp and Rubisco proteins, their expressions were higher than that of the other genes.

In the following sections, we discuss in greater detail individual metabolic pathways that are significantly affected by albino and pale green mutations, focusing on the roles of the genes responsible for the respective mutations as proteins in chloroplast.

### Promotion of nitrogen assimilation

**Fig. 5:**
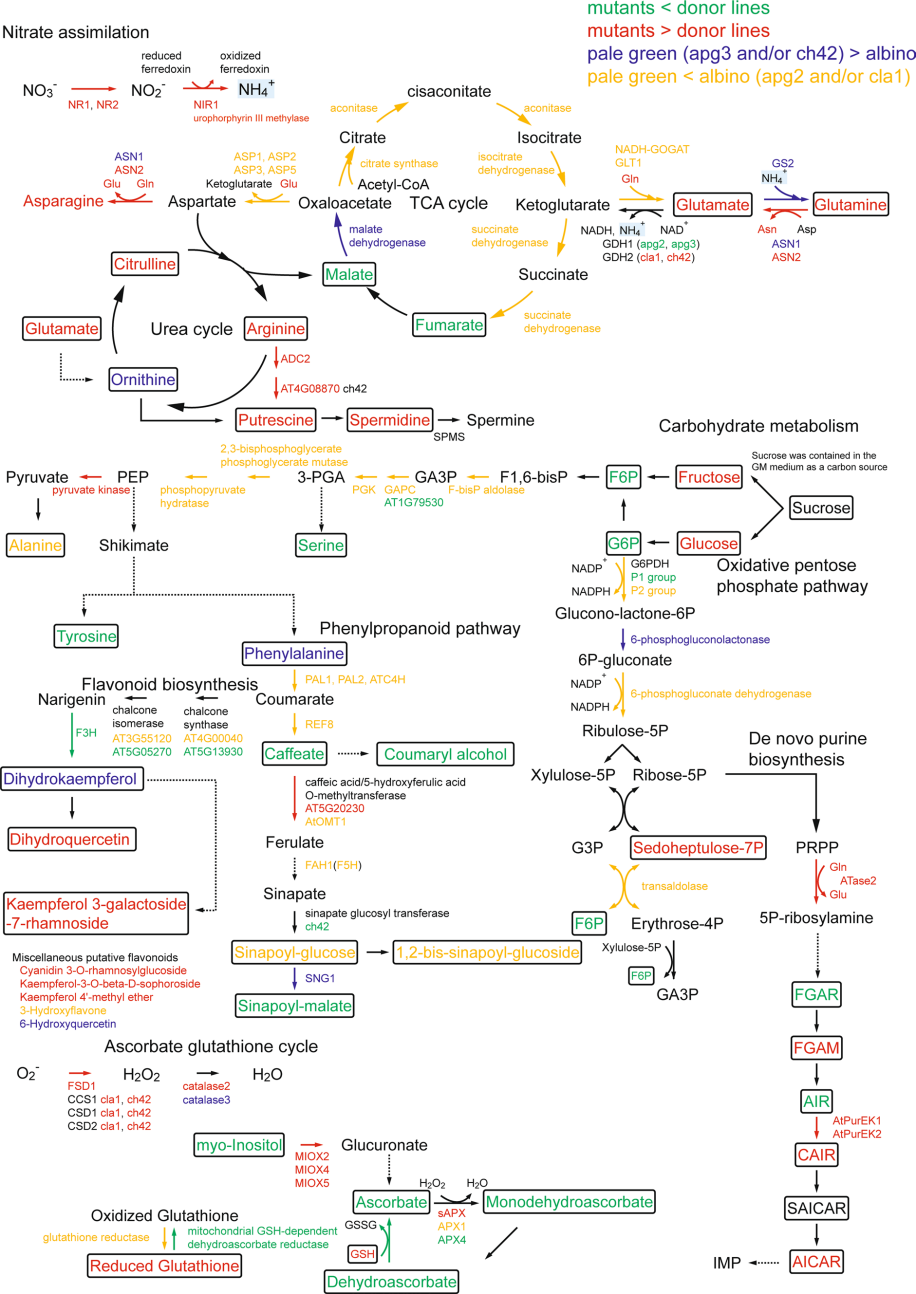
The scheme of metabolic pathways determined using metabolome analysis and transcriptome analysis. *Boxed* compounds indicate metabolites detected by the FT-ICR/MS or GC-TOF/MS. *Solid arrows* connected by compounds indicate an enzyme reaction. *Dashed lines* indicate abbreviation of several enzyme reactions. *Colors* of compounds and enzyme reactions indicate the relative abundance as indicated by mass spectrometers and gene expression by microarray, respectively. The following color codes are used: green, reduction of transcripts and metabolites in the mutants rather than *Ds* donor lines; *red*, accumulation of transcripts and metabolites in the mutants rather than *Ds* donor lines; *blue*, accumulation of transcripts and metabolites in the pale-green mutants (*apg3*, *ch42*) rather than albino mutants (*apg2*, *cla1*) or accumulation only in the *ch42* mutant; *yellow*, accumulation of transcripts and metabolites in the albino mutants (*apg2*, *cla1*) rather than pale-green mutants (*apg3*, *ch42*) or accumulation only in the *cla1* mutant

Microarray analysis of the albino and pale green mutants revealed the most affected genes in metabolic pathways were those associated with nitrate assimilation. Genes encoding enzymes of nitrate assimilation have been reported (Wang et al. 2003). Of the 2,812 altered genes, six of these genes (Supplemental Table 4) were strongly up-regulated. These genes included one in the pathway leading from nitrate to ammonium, a nitrate transporter, a nitrate reductase (NR1, NR2), a ferredoxin-nitrite reductase (NiR1) (Fig. 5, top left red arrows; Supplemental Fig. 3) and an uroporphyrin III methylase that works as coenzyme for NiR1. The gene expressions of ferredoxin required for NiR1 and ferredoxin- NADP reductase were up-regulated. As previously reported (Wang et al. 2003), nitrate induced the expression of nicotianamine synthase. The up-regulation of the nicotianamine synthase gene was also observed in the mutants. Therefore, the mutants appear to assimilate nitrate into ammonium.

The assimilated ammonium is incorporated into the GS/GOGAT cycle (glutamine synthetase/glutamine-oxoglutamate aminotransferase) and synthesised into glutamine and glutamate. Asparagine is synthesised by asparagine synthetase (ASN) transforming glutamine into glutamate (Oliveira and Coruzzi 1999; Weber and Flügge 2002). GS2 was up-regulated in the *cla1* and *apg2* mutants, and GLT1 (NADH-GOGAT) was slightly up-regulated in the *cla1* mutant. Among asparagine synthesis enzymes, ASN1 was up-regulated only in *ch42* and ASN2 was up-regulated in all mutants. ASN2 may play an important role in the re-assimilation of ammonium derived from protein degradation under stress conditions (Wong et al. 2004). The gene expressions of four aspartate aminotransferases involved in aspartate synthesis, ASP1, ASP2, ASP3 and ASP5, were strongly up-regulated in the *cla1* mutants and slightly up-regulated in the *apg2* and *apg3* mutants, but were unchanged in the *ch42* mutants. In the mutants, accumulation of glutamate, glutamine, and asparagine was confirmed by metabolome analysis using FT-ICR/MS and GC-TOF/MS. Therefore, it is possible that the pathways in which ammonium is synthesized into amino acids via nitrate assimilation are activated in the mutants.

### TCA cycle and glycolysis pathway

The most affected mutant in the expression of TCA cycle genes was *cla1* (Supplemental Table 5). The expression levels of genes in the pathway from citrate to fumarate were highly up-regulated in the *cla1* mutant (Fig. 5, top TCA cycle, yellow arrows; Supplemental Fig. 3). In contrast, malate dehydrogenase was down-regulated in the *cla1* mutant (Fig. 5, top TCA cycle, green arrows; Supplemental Fig. 3). GC-TOF/MS analysis revealed that amounts of fumarate and malate were reduced in the mutants, particularly in the *apg2* and *cla1* mutants that had an albino phenotype (Supplemental Table 2). It has been reported that in nitrogen-starved tobacco plants, the addition of either nitrate or ammonium increased the gene expression of isocitrate dehydrogenase, aconitase and citrate synthase in the TCA cycle, to transform assimilated nitrogen into amino acids. In the GS/GOGAT cycle, two glutamates are synthesized from 2-oxoglutarat via transamidation. One of the glutamates becomes glutamine, while the other is used for other biosynthetic reactions (Lancien et al. 1999). Therefore, it is possible that the reason the TCA cycle is activated in the *cla1* mutant is to provide the GS/GOGAT cycle with 2-oxoglutarate for re-assimilated ammonium.

Expressions of glycolysis genes were up-regulated in the *cla1* mutant and slightly up-regulated in the *apg2* and *apg3* mutants, but were unchanged in the *ch42* mutant (Supplemental Table 5; Fig. 5, center of carbohydrate metabolism; Supplemental Fig. 3). These gene expression profiles correlate with the position of the mutants on the PC2 axis in the PCA results of FT-ICR/MS (positive mode) (Fig. 2), and also with the degree of disrupted chloroplast inner membranes in the mutants (Fig. 1). Thus it is suggested that the more chloroplast inner membranes are disrupted, the more glycolysis gene expression is activated. These results should be reflected in the amount of metabolites; GC-TOF/MS analysis revealed that fructose and glucose were accumulated in the mutants. It is noted that soluble sugars such as fructose, glucose and sucrose are associated with oxidative stress (Couée et al. 2006). It is surprising that these sugars were accumulated in mutants that cannot fix carbon dioxide due to the repressed expression of photosynthesis genes. This phenomenon may have occurred as a result of oxidative stress, such as the accumulation of reactive oxygen species (ROS).

### Oxidative pentose phosphate pathway

The oxidative pentose phosphate pathway (OPPP) is a major pathway that generates reducing power like that of NADPH in non-photosynthetic tissues and photosynthetic tissues in the dark (Emes and Neuhaus 1997). The main regulatory enzymes of OPPP are glucose 6-phosphate dehydrogenase (G6PDH) and 6-phosphogluconate dehydrogenase (6PDGH). The OPPP acquires two molecules of NADPH upon transforming from glucose 6-phosphate into ribulose 5-phosphate (Fig. 5, center right; Supplemental Fig. 3). The generated reducing power is used as a major cofactor of ROS scavenging pathways such as ascorbate–glutathione cycles (Couée et al. 2006), and used for nitrate assimilation by ferredoxin- NADP reductase. The G6PDH enzymes are divided into a chloroplastic isoform (P1 group) and a plastidic isoform (P2 group) based on their physiological roles (Wakao and Benning 2005). The P1 group of G6PDH is expressed in the developing tissues except for roots, and is tightly regulated by the redox potential (NADPH/NADP+ ratio) in photosynthesis. On the other hand, those of the P2 group are highly expressed in the root and are more resistant to NADPH levels. The reducing power in the root is linked to nitrogen assimilation (Neuhaus and Emes 2000; Esposito et al. 2005). Of the three G6PDH enzymes, the expression of the P1 group enzyme AT5G35790.1 was down-regulated in all mutants, however the expressions of the P2 group enzymes AT5G13110.1 and AT1G24280.1 were up-regulated all but the *ch42* mutant (Supplemental Table 5). The reason for the down-regulation of P1-G6PDH could be the lowering of the NADPH/NADP+ ratio due to lost photosynthetic ability (Fig. 1). It is thought that heterotrophic albino mutants using the nitrate in the GM medium are actively performing nitrate assimilation. The reason for the up-regulation of P2-G6PDH could be the production of the reducing power required for nitrate assimilation.

### De novo biosynthesis of purine nucleotides

AMP and GMP (de novo biosynthetic products of the purine nucleotide pathway) are fundamental to the structure of DNA and RNA, as well as for a number of essential coenzymes such as NAD, NADP, and FAD. The amounts of intermediate metabolites in this pathway were strongly affected in the mutants as indicated by FT-ICR/MS analysis (Fig. 5, bottom right). Furthermore, the expression of PRPP amidotransferase 2 (ATase2), a rate-limiting step in this pathway, was confirmed by microarray analysis to be up-regulated in the mutants (Supplemental Table 5). These results suggested that the synthesis of purine nucleotides was activated in the mutants. One possibility is that the strong accumulation of glutamine in the mutants could activate this pathway. In nodules of N-fixing tropical legumes, such as soybean (*Glycine max*) and cowpea (*Vigna unguiculata*), fixed N is assimilated initially through the amide group of glutamine, and the majority of it is subsequently incorporated through this pathway to form IMP (Smith and Atkins 2002). Thus, strong accumulation of glutamine in the mutants might be regulating the activity of this pathway. Another possibility is that this pathway is involved in chloroplast biogenesis in some way. The de novo biosynthesis of purine nucleotides, starting from glutamine and PRPP (5-phosphoribosyl diphosphate) that are synthesized from ribose-5P, could be considered to be localized at chloroplast, because sequences of their related enzymes are predicted to encode N-terminal plastid-transit peptides (Zrenner et al. 2006). It has been reported that the *cia1* mutant that is disrupted in the PRPP amidotransferase 2 gene committing step in de novo purine synthesis shows albino pale-green mosaic leaves, and reduced import efficiency of proteins into chloroplasts (Hung et al. 2004). Because the *cia1* mutant plants have very low levels of ATP and GTP, a decrease in energy may be altering growth and chloroplast protein import significantly. A third possibility is that its role in recycling and remobilization and purine catabolism might be involved in plant response and adaptation to environmental stress. 2-azahypoxanthine (AHX) is a compound responsible for the fairy-ring phenomenon caused by fungus. AHX chemically synthesizes from 5-aminoimidazole-4-carboxamide (AICA), and AICA is one of the members of the purine metabolic pathway. AHX and its metabolite (2-aza-8-oxohypoxanthine) are thought to be produced by plants themselves through a pathway similar to chemical synthesis (Choi et al. 2010; Choi et al. 2014). They stimulate plant growth (Choi et al. 2014; Tobina et al. 2014) and give environmental tolerance (unpublished data). Moreover, the purine metabolite allantoin enhances abiotic stress tolerance (Watanabe et al. 2014). For the same reason, the albino and pale green mutants may be trying to activate this pathway in order to gain energy, reclaim chloroplast functions and protect against environmental stress.

### Ascorbate–glutathione cycle and excess energy removal

Expressions of several genes related to the removal of excess energy changed between the four mutants and their controls. There were eight genes affected in the ascorbate–glutathione cycle (Chew et al. 2003), six genes among superoxide dismutase (SOD) and catalase, and three genes encoding myo-inositol oxygenase that are related to the synthesis of ascorbate (Lorence et al. 2004) (Supplemental Table 5; Fig. 5, bottom left; Supplemental Fig. 3). In particular, clearly up-regulated genes in all mutants were the chloroplast Fe superoxide dismutase (AT4G25100) gene, a stromal ascorbate peroxidase (AT4G08390) gene, and genes encoding three myo-inositol oxygenases, MIOX2 (AT2G19800), MIOX4 (AT4G26260), and MIOX5 (AT5G56640). These results suggested that the albino and pale green mutants have been required to scavenge generated ROS. Nevertheless, the metabolome analysis showed the strong reduction in amounts of ascorbate, monodehydroascorbate, and dehydroascorbate; compounds related to xanthophyll cycle such as zeaxanthin, violaxanthin (Gilmore and Yamamoto 1993); and alpha-tocopherol, a compound which functions as the antioxidant tocopherol (Fryer 1992) (Supplemental Table 1). Because terpenoids including xanthophyll are synthesized in chloroplast, the albino and pale green mutants that repressed photosynthesis genes were assumed to decrease the synthesis rates of these compounds. Therefore, we suggest that the functions of the ascorbate–glutathione cycle and xanthophyll cycle are not sufficient to remove ROS in the albino and pale green mutants.

Accumulation of pyridoxine (Vitamin B_6_) and its derivatives, glucosylpyridoxine and pyridoxate were detected by FT-ICR/MS (Supplemental Table 1). Pyridoxine has been reported to play a role in protecting membranes from lipid peroxidation (Chen and Xiong 2005). Synthesis of pyridoxine and its active form pyridoxal 5-phosphate start from glutamine and ribose 5-phosphate. Expressions of two genes related to this pathway PDX1.1 (AT2G38230) and PDX1.3 (AT5G01410) (Wagner et al. 2006) were slightly up-regulated (Supplemental Table 5). It is possible that the accumulation of glutamine activates the down-stream events of this pathway in the mutants, similar to the activation of the de novo purine biosynthesis pathway.

### Phenylpropanoid biosynthesis pathways

The expression profiles of genes related to phenylpropanoid biosynthesis correlated with the position of the mutants along the PC2 axis in the PCA results of FT-ICR/MS (positive mode), which is the same as that of OPPP and glycolysis (Supplemental Table 5; Fig. 3 center). These results suggested that the disruption of the chloroplast inner membrane influenced the synthesis of aromatic secondary metabolites via the phenylpropanoid biosynthesis pathway. FT-ICR/MS analysis showed that five phenylpropanoids were detected to have different relative abundance when compared between the mutants and their *Ds* donor lines. Sinapoyl-glucose and bis sinapoyl-glucoside were accumulated in the *cla1* and *apg2* mutants whereas they were reduced in the *ch42* mutant. Sinapoyl-malate, caffeate and coumaryl alcohol were reduced in all of the mutants (Supplemental Table 1). Three sinapoylesters, sinapoyl-malate, sinapoyl-glucose and sinapoyl-choline have been found predominantly in plants in the Brassicaceae family (Lorenzen et al. 1996), and each sinapoylester is utilized at different times in the course of plant development (Ruegger et al. 1999). Sinapoyl-malate plays a role in UV-B protection (Booij-James et al. 2000) and is synthesized from sinapoyl-glucose by a sinapoyl-glucose:malate sinapoyltransferase; however it is not synthesized in seedlings in the dark. Thus, the accumulation of sinapoyl-malate requires light factors (Ruegger et al. 1999). Sinapoyl-glucose and bis sinapoyl-glucoside had a greater accumulation in the *cla1* mutants without chloroplast inner membranes. In contrast, the smallest level of reduction of sinapoyl-malate was in the *ch42* mutant. It is possible that the reason sinapoyl-glucose accumulates is due to the fact that *cla1*, not having pigment and not being able to detect light, cannot use sinapoyl-glucose:malate sinapoyltransferase to synthesize Sinapoyl-glucose to sinapoyl-malate.

### Inconsistencies in metabolome and transcriptome states of each albino and pale green mutant

Our results show that there are partial inconsistencies between metabolic and transcriptomic states in albino and pale green mutants. The *apg2* and *cla1* mutants have the same albino appearance but have different metabolic phenotypes, and the *apg2* mutant is more similar to the *apg3* mutant which has a pale-green appearance, than to the *cla1* mutant. The *cla1* mutant is the most unique among the four mutants, because it lacks chlorophylls and carotenoids. The effect of the *ch42* mutation was relatively light compared with the other mutants. The reason for this is possibly that the gene mutated in *ch42* is one of two genes that make up the Mg-chelatase subunit CHLI, therefore the only direct effect is a reduction in chlorophyll content. Between the *apg2* and *apg3* mutants, the profiles of gene expression and metabolic distribution were similar. The reason for this similarity is that the biochemical changes of these mutants caused by their mutations are equivalent to the down-regulation of photosynthesis-related genes. The *APG2* gene encodes a component of the delta pH-dependent protein transporter in plastids, and the mutation in the gene causes a reduction of thylakoid membrane proteins such as D1, light-harvesting complex and OE23 (Motohashi et al. 2001). The *APG3* gene is thought to be associated with the termination of plastid protein translation (Motohashi et al. 2007). From these results it is thought that the changes in transcriptome and metabolome data in *apg2* and *apg3* may be caused by the decrease of plastid proteins.

Although phenotype is similar in *cla1* and *apg2* mutants (neither has thylakoid at all in their plastids), the results of their transcriptome and metabolome analysis are not identical. The results of these omics analyses reflects the influence of the defect each present in gene.

### The usefulness of integrated analysis of metabolome and transcriptome in order to understand chloroplast functions

We used two types of mass spectrometry systems, FT-ICR/MS and GC/MS. GC/MS is a standard system to analyze various metabolites in plants. By using Infusion ESI FT-ICR/MS, we can discriminate among mutants that show albino or pale-green phenotypes based on the relative abundance of metabolites, which concludes that FT-ICR/MS is an appropriate method for metabolome analysis. The ChloroP software that predicts chloroplast transit peptides indicated that approximately 2,100 proteins may be localized in plastids (Richly and Leister 2004). By comprehensively analyzing the mutants of those genes, we will be able to better understand the relationship between phenotypes (albino, pale-green, and silent phenotypes like wild-type) and the genes responsible, which may offer clues to understanding novel chloroplast systematic mechanisms. The analysis of albino mutants using one- and two- dimensional nuclear magnetic resonance spectroscopy has been reported (Tian et al. 2007). NMR can be used to analyze metabolome in planta. However, mass spectrometry systems detect metabolites at a much higher sensitivity than NMR.

## Conclusion

The comparative transcriptome and metabolome analysis of the four albino mutants in comparison to control *Ds* donor plants revealed a common molecular and metabolite phenotype for photosynthesis-impaired mutants. The high number of similar gene expressions and metabolites is surprising because we used albino mutants that have a lack of function of different genes. Because of their similar phenotypes (phenome), we presumed correctly that their metabolomes would be similar to their phenomes, but what was interesting was that the transcriptomes of each mutant were also quite similar.

At same time we were also able to find characteristics specific to each mutant. In the future, when metabolome analysis techniques have advanced and we can quantitatively detect more metabolites in plants, we can obtain more useful mutation-specific data to further analyze gene functions.

## Materials and methods

### Plant material and growth conditions

Four *apg* mutants were isolated from transposon-tagged lines that were crosses between transgenic lines expressing *Ac* transposase in the female parent and *Ds*-GUS-T-DNA lines (*Ds*54: *Ds389*-13, *Ds*52: *Ds391*-20, *Ds*53: *Ds392*-13, *Ds*13: *Ds3*-390-1) in pollen parents (Ito et al. 2002, 2005; Kuromori et al. 2004; Myouga et al. 2010). The plants were grown on germination medium (GM) containing Murashige and Skoog salts and 1 % sucrose (Wako, Osaka, Japan), for 3 weeks in a growth chamber maintained at 22 °C, with 16 h light and 8 h dark cycles. In order to equalize the biological bias, the mutants and their *Ds* donor lines as controls were sown in the same plates and rotated every 3 days. Pooled samples of equally volume were gathered in the same manner from more than 15 dishes. Samples used for microarray analysis were whole tissues. For metabolome analysis, after removing roots, samples were frozen in liquid nitrogen immediately and stored at −80 °C until use.

### Electron microscopic analysis

Leaves were fixed, ultrathin sections were cut and stained, and electron microscope (Jeol, Tokyo, Japan) observations were made according to protocol in Motohashi et al. (2001).

### Chlorophyll fluorescence measurements

Chlorophyll fluorescence of leaves was measured at room temperature using a pulse-amplitude-modulated (PAM) fluorometer (TEACHING-PAM, Walz, Effeltrich, Germany) and a photosynthesis yield analyzer (MINI- PAM, Walz, Effeltrich, Germany). Before the chlorophyll fluorescence measurements, plants grown normally for 3 weeks were dark-adapted for 20 min. The results represent the mean values of at least four measurements.

### Pigment analysis

Pigment extraction, analysis, and quantification were performed according to protocol in Iuchi et al. (2001).

### Metabolome analysis using infusion ESI FT-ICR/MS

After the samples were homogenized with liquid nitrogen, the powdered samples were dissolved in extraction solvents to 20 % fresh weight/volume. Two extraction solvents, acetone and methanol, were used to elute various polar compounds. Extracted sample solutions were filtered through 0.4 µm filters and dissolved in 10 % (v/v) H_2_O: methanol:acetone (4:4:2). Mass analysis using FT-ICR/MS (Apollo II ESI Apex-Q70e, Bruker Daltonics, Billerica, MA, USA) was performed in positive and negative ionization operation modes. For positive and negative mode, acetic acid was added to extracted sample solutions at a final concentration of 2 % (v/v) and ammonium acetate added at 0.1 % (v/v). Mass spectra were acquired over the 100–1,000 m/z range and accumulated to improve the S/N ratio. We measured the product ion mass spectra of each sample three times. When ion peaks were detected at least twice out of three successive spectral scans, they were subjected to further data processing as ion signals from actual analytes. A total of four mass spectral peaks from two different extraction solvents (methanol and acetone) and two ionization operation modes (positive and negative) were aligned with our original Java program.

Following this, we applied global normalization to our data. To avoid zero division, missing values were filled with 10^5^ as a background signal of FT-ICR/MS. Peak intensities were transformed using a logarithmic scale with a factor of 10. Four data matrices were used to apply global normalization. Global normalization calculation methods are as follows: (1) Average intensity was calculated by dividing the total signal by the number of detected peaks in each spectrum. (2) Average signal was calculated for all spectra in each elution and charge pair. (3) Normalization factor was calculated for each spectrum by dividing the average intensity for each spectrum by total average intensity. (4) Normalized intensity was calculated by multiplying the raw intensity in each spectrum by the previously calculated normalization factor. Empirical formulas were inferred by the accuracy of the FT-ICR/MS. Because the sample ions became adduct ions to attach proton and sodium ion, etc. on the ESI source, we assumed the following were involved in the detected peaks [M+H]+, [M+Na]+, [M+K]+, [M+H+methanol]+, [M+ammonium]+ in the positive ionization mode and [M−H]− in the negative ionization mode. We searched for candidate compounds using KEGG (http://www.genome.jp/kegg/), NIST (http://webbook.nist.gov/chemistry/) and KNApSAck (Shinbo et al. 2006).

### Metabolome analysis using GC-TOF/MS

Six replicate samples, mutants and their *Ds* donor lines were obtained from unique dishes. Metabolites were extracted from 50 mg of frozen plant material with CHCl_3_:MeOH:H_2_O (2:6:2) that included 10 stable isotope reference compounds. Metabolites were derivatized by methoxyamination for 22 h, using a 20 mg/ml solution of MeONH_3_ in pyridine. The derivatives were subsequently trimethylsilated with *N*-methyl-*N*-trimethylsilyl-trifluoacetamide for 1 h. An n-heptane mixture (30 μl) was used for the determination of retention time indices. Samples were injected in splitless mode (2 μl/sample) and analyzed using GC-TOF/MS (Pegasus 4D; Leco, St. Joseph, MI, USA). Artifact peaks were manually identified and removed from the obtained peaks. Remaining peaks in each mass spectrum were transformed using × 1,000 and + 1 in order to form a normal distribution. Peak intensities were transformed using a logarithmic scale with a factor of 10.

### Principal component analysis and t-test

Statistical analyses, including principal component analysis (PCA), *t* test and regression plots were performed in an R software environment for statistical computing version 2.0.1; http://www.r-project.org/ (Ihaka and Gentleman 1996). To calculate eigenvalue, the eigenvector for PCA, *prcomp* in R function was implemented with a correlation matrix. The contribution ratio (%) in each component was the eigenvalue divided by total number of peaks in a matrix, ×100. The component loadings in each peak were the square root of the eigenvalue multiplied by the eigenvector. Principal component scores were obtained from *prcomp*, the variance of which was adjusted to be the eigenvalue.

LogRatios for each peak were calculated by the weighted mean of the mutant log transformed intensities minus that of the *Ds* donor line. One-step Tukey’s Biweight Estimate was adopted as a weighted mean method, which is known as a robust method against outliers in microarray intensity calculation. *P* value was calculated by Welch’s t-test and adjusted to controlled probability using Q-VALUE software (Storey and Tibshirani 2003) in consideration of the multiple comparisons problem.

### Transcriptome analysis using Agilent Arabidopsis2 Oligo Microarray

Total RNA was isolated using Trizol according to the supplier’s instructions (Life Technologies, Rockville, MD, USA) and purified with an RNeasy Plant Mini Kit (Qiagen). cDNA was synthesized with 700 ng of total RNA using MMLV-RT and T7 promoter primers (Agilent Technologies, Inc., Palo Alto, CA, USA). This cDNA was used as a template to synthesize cRNA. The cRNA was labeled with Cyanine-3 (Cy3) CTP or Cyanine-5 (Cy5) CTP (Perkin Elmer/NEN Life Sciences, Tokyo, Japan). Cy3-labeled cRNA (700 ng) was mixed with the same amount of Cy5-labeled cRNA and used for subsequent hybridization. After hybridization for 17 h at 60 °C, slides were washed and scanned with an Agilent Microarray Scanner. Scanned images were transformed into quantified figures by the Agilent Features extraction software (version 7.1). We attempted dye swap experiments to estimate for human error and the consistency of the dye labeling.

### Data mining of microarray results for transcriptome analysis

Unreliable spots at four filters (IsSaturated, IsWellAboveBG, IsFeatureNonUnifOL, IsPosAndSignif) were excluded from subsequent analyses. We attempted to detect the spots that have statistically significant differences in signal intensity, based on the PValueLogRatio calculated from the Feature Extraction software; this indicates the level of significance in the differential expression of a gene as measured through the log ratio. Then the PValueLogRatios, including color swap replicates, were corrected to the q-value (Storey and Tibshirani 2003) and chosen when FDR < 10^−4^. The list of significant genes in all experiments was partitioned into clusters based on expression similarity using Cluster software (Eisen et al. 1998). The similarity metric was determined using Correlation (uncentered), calculation of centroids was carried out using Average Linkage, and we used Java TreeView to create the GeneTree (Saldanha 2004). Eight distinctive clusters were isolated. Functional categories of the genes in these clusters were determined on the basis of Gene Ontology and GO Slim (ATH_GO_GOSLIM.20060422) in TAIR (Rhee et al. 2003). As reported by Zeeberg et al. (2003), one-sided Fisher’s exact tests were performed. The null hypothesis was that the GO category is not enriched in cluster genes compared to what would have been expected by chance alone.

### Ammonium analysis

NH_4_^+^ content was determined in whole 3-week plants crushed in liquid nitrogen. Ammonium extraction, purification and quantification were performed according to protocol in Bräutigam et al. (2007). We used a kit (modified Fujii-Okuda method, Wako) as the Berthelot reaction for the quantification of ammonium. NH_4_^+^ concentration was determined using an NH_4_Cl standard curve, normalized to gram fresh weight of the sample.

## Electronic supplementary material

Below is the link to the electronic supplementary material.

Supplementary material 1 (XLS 80 kb)

Supplementary material 2 (XLS 19 kb)

Supplementary material 3 (XLS 1229 kb)

Supplementary material 4 (XLS 41 kb)

Supplementary material 5 (XLS 41 kb)

Supplementary material 6 (PDF 544 kb)

Supplementary material 7 (PDF 448 kb)

Supplementary material 8 (PDF 1642 kb)

## Abbreviation

WT: Wild type
