# Supplementary material for: Integrated analysis of transcriptome and metabolome of Arabidopsisalbino or pale green mutants with disrupted nuclear-encoded chloroplast proteins
Source: Plant Mol Biol. 2014 May 3;85(4):411–28. doi: 10.1007/s11103-014-0194-9 (PMC4052017; doi:10.1007/s11103-014-0194-9)
Supplement: Supplementary file 6 — Supplementary material 6 (PDF 544 kb) [file 11103_2014_194_MOESM6_ESM.pdf]

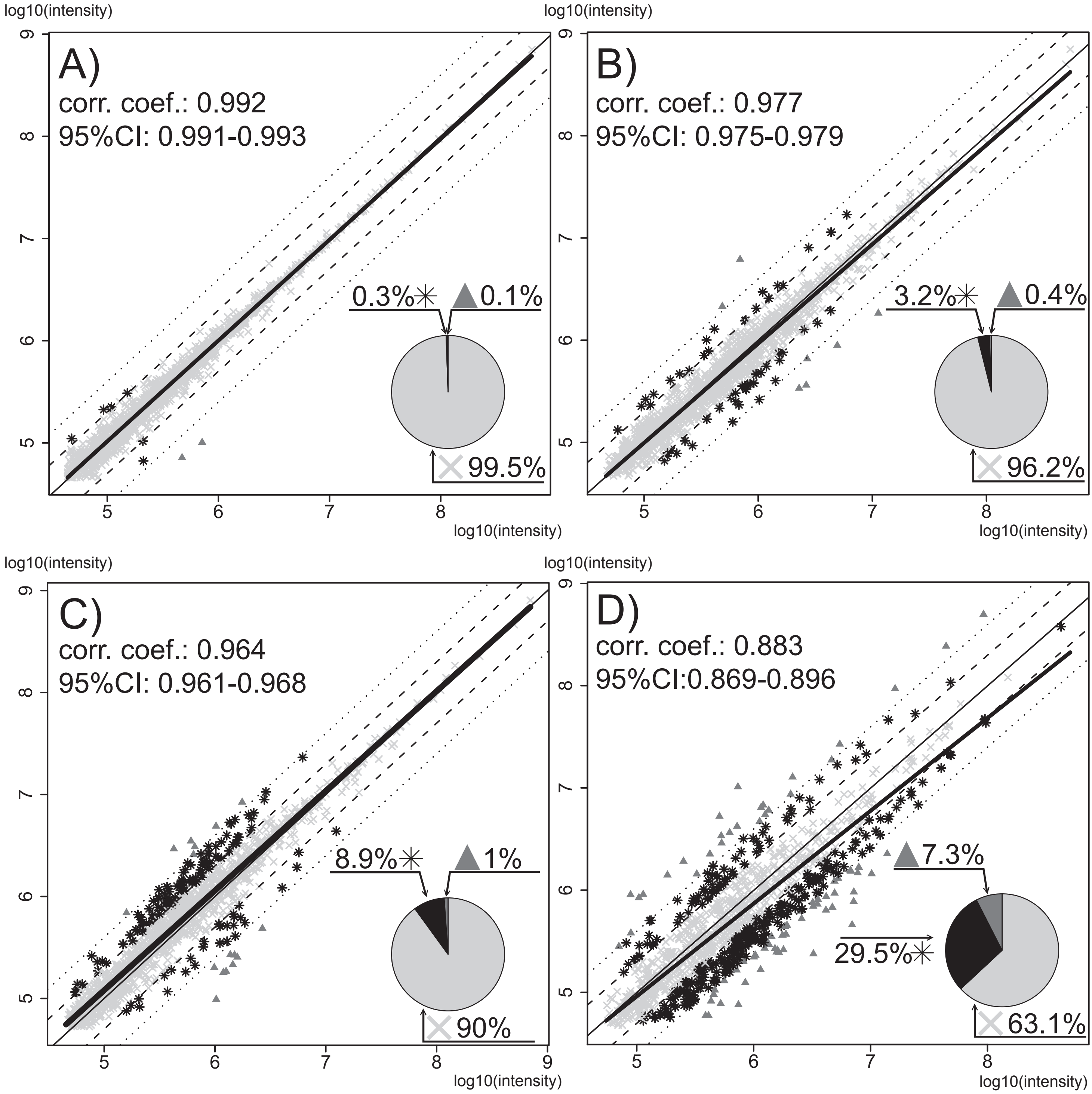

Supplemental Figure 1. Scatter plots showing the reproducibility of the FT-ICR/MS peak intensity. On the top left corner of each panel, Pearson's correlation coefficients (corr. coef.) and its 95% confidence interval are indicated. Narrow solid lines have a slope of 1. Thick lines are fitted to the data points by means of least square linear regression. Long-dashed lines have slopes from +0.3 to -0.3. Short-dashed lines have slopes from +0.6 to -0.6. Cross symbols of the data points indicate that the range of log ratio is from -0.3 to +0.3. The asterisk symbols indicate ranges from -0.6 to -0.3 or from +0.3 to +0.6. The filled triangle symbols indicate ranges over +0.6 or under -0.6. Pie graph at the bottom left corner of each panel indicates the proportion of data points. (A) Scatter plot showing two measurements from the same extraction solution. Pearson correlation of coefficient was 0.992 (95% confidence interval, 0.991-0.993), indicating that spectral scan data from the same sample were reproducibly obtained under our experimental conditions. (B) In order to examine whether or not sample preparation processes might affect the reproducibility in the MS analyses, we analyzed extracts prepared independently from several seedling pools from a single culture dish as technical replicate experiments.Data spread was greater in (B) than in (A), however, only 3.6% peaks exhibited intensity fluctuation greater than +/-0.6. These results indicated that our FT-ICR/MS analyses were done in a reproducible manner in terms of analytical repetition and sample extraction. (C) Results of different extraction solutions, x-axis, methanol-extracted; y-axis, acetone-extracted. The correlation was 0.964. Compared with (B) and (C), the dots in the intensity range of 5 to 6.5 were extended. These results demonstrated that barely extractable analytes by a given solvent could be efficiently extracted with another solvents. (D) Comparison of the mutant (cla1) to the donor line using the same extraction method. Unlike the three scatter plots (A), (B), (C) described previously, peaks that were more than +0.6 or less than -0.6 were increased remarkably in this data. This indicating clearly different metabolic conditions between the cla1 mutant and the cla1 Ds donor line.
