# Supplementary material for: Integrated analysis of transcriptome and metabolome of Arabidopsisalbino or pale green mutants with disrupted nuclear-encoded chloroplast proteins
Source: Plant Mol Biol. 2014 May 3;85(4):411–28. doi: 10.1007/s11103-014-0194-9 (PMC4052017; doi:10.1007/s11103-014-0194-9)
Supplement: Supplementary file 7 — Supplementary material 7 (PDF 448 kb) [file 11103_2014_194_MOESM7_ESM.pdf]

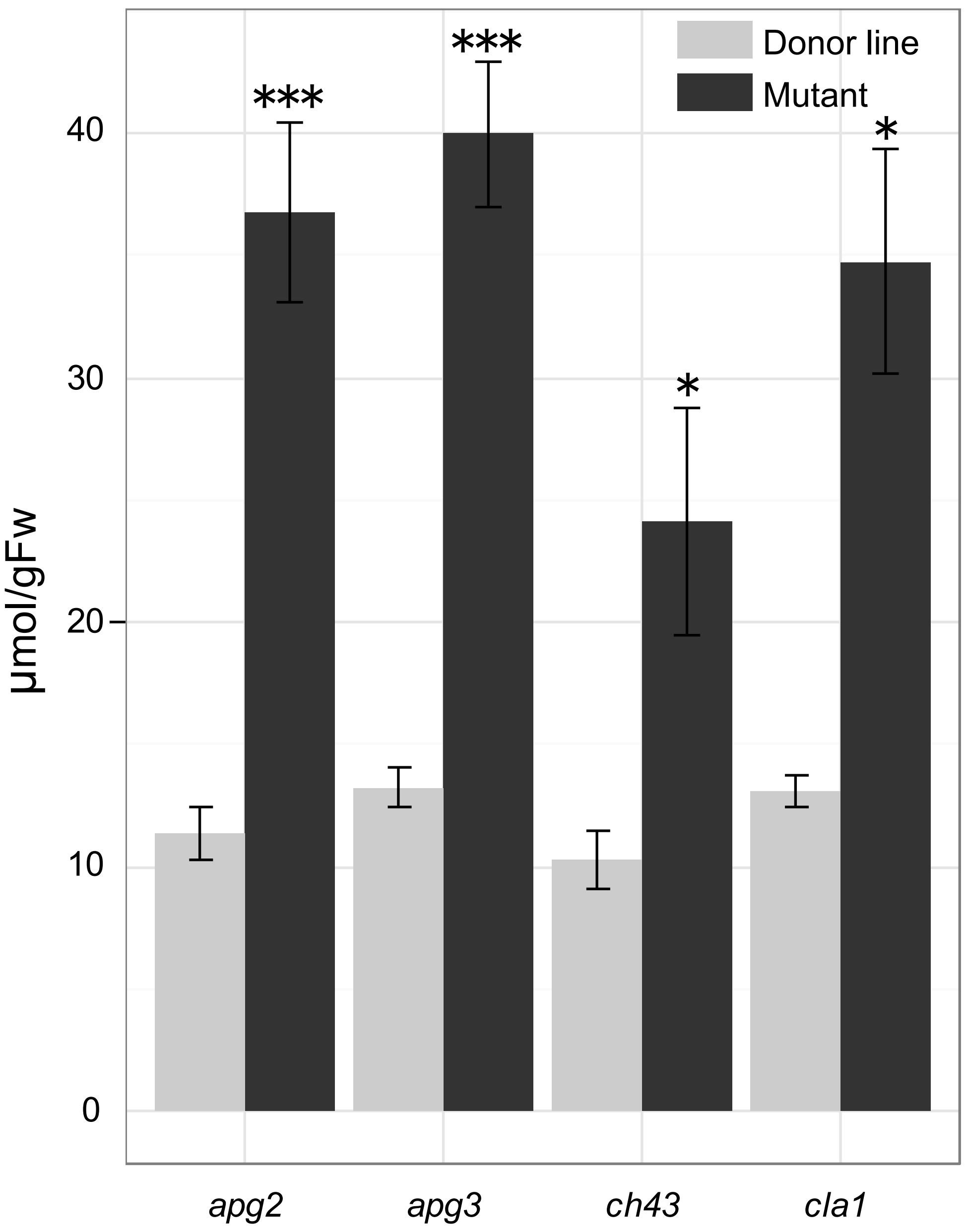

Supplemental Figure 2. Endogenous ammonium levels in albino and pale green mutants and their *Ds* donor lines (control). Bar graph values are μ mol normalization to per gram fresh weight of the sample. The error bars represent the standard deviation (n=3 biological replicate experiment). Asterisks denote statistical significance compared to *Ds* donor lines (*Weltch't-test: P<0.05, ***P<0.01).
