# Supplementary material for: Integrated analysis of transcriptome and metabolome of Arabidopsisalbino or pale green mutants with disrupted nuclear-encoded chloroplast proteins
Source: Plant Mol Biol. 2014 May 3;85(4):411–28. doi: 10.1007/s11103-014-0194-9 (PMC4052017; doi:10.1007/s11103-014-0194-9)
Supplement: Supplementary file 8 — Supplementary material 8 (PDF 1642 kb) [file 11103_2014_194_MOESM8_ESM.pdf]

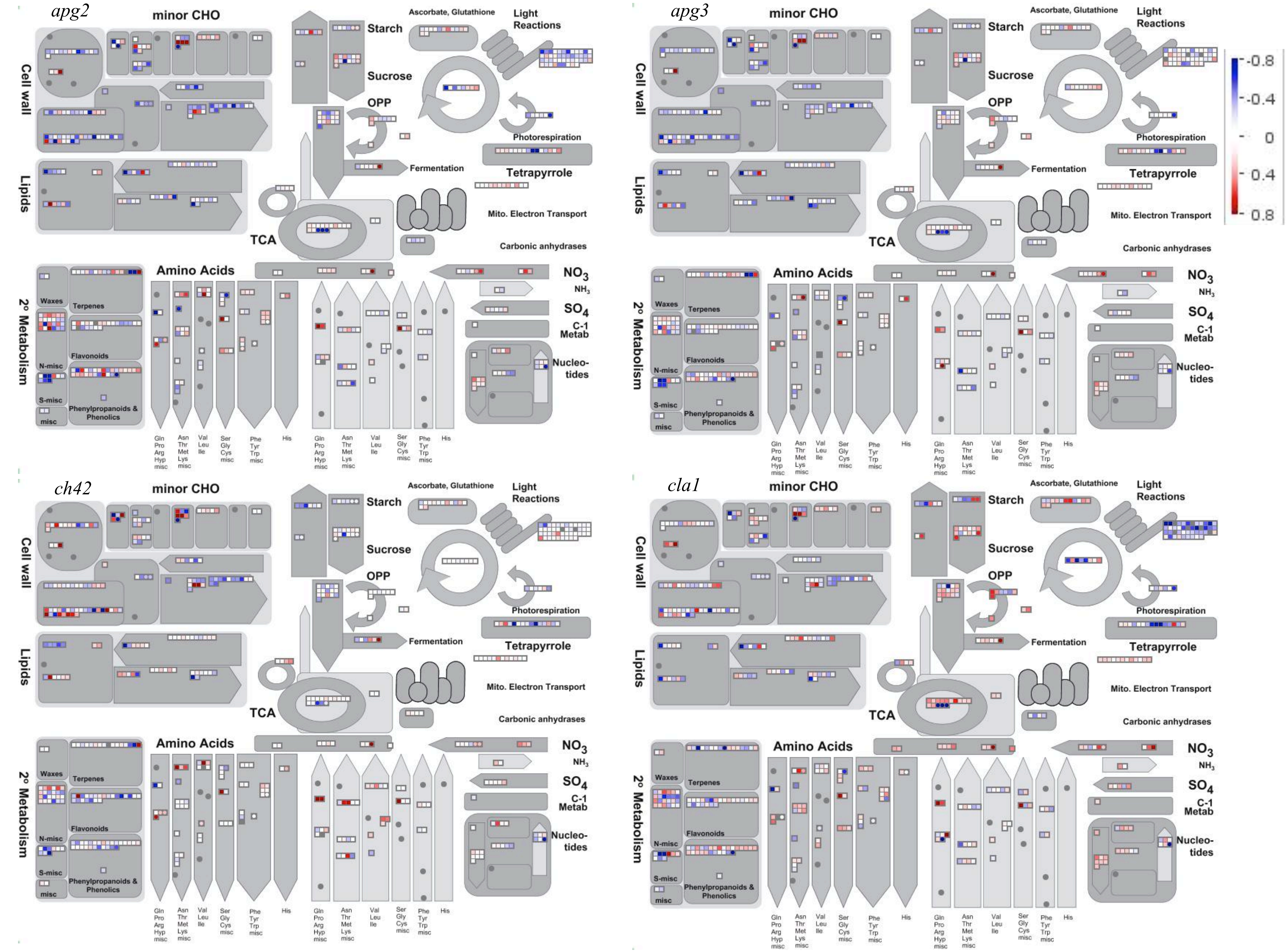

Supplemental Figure 3. A MapMan overview of the transcripts and metabolites which have a function in metabolism that show up-regulation (red) or down-regulation (blue) in each of the albino mutants compared with their respective *Ds* donor lines. Data from Supplemental Table 3 was used to make this diagram. Metabolites were detected using FT-ICR/MS and GC-TOF/MS. Metabolites detected with adduct ions were also included, taking an average of all amounts detected. Transcripts are represented by a □, while metabolites are represented by a ○. CHO = Carbohydrate; OPP = Oxidative pentose phosphate; TCA = Ttricarboxylic acid.
